# Alpha-Fetoprotein Regulates the Expression of Immune-Related Proteins through the NF-*κ*B (P65) Pathway in Hepatocellular Carcinoma Cells

**DOI:** 10.1155/2020/9327512

**Published:** 2020-07-28

**Authors:** Qiu-ting Li, Meng-jun Qiu, Sheng-li Yang, Xiefan Fang, Xiao-xiao He, Meng-meng Wang, Ya-nan Li, Zhi-fan Xiong, Shanshan Huang

**Affiliations:** ^1^Division of Gastroenterology, Liyuan Hospital, Tongji Medical College, Huazhong University of Science and Technology, Wuhan 430077, China; ^2^Cancer Center, Union Hospital, Tongji Medical College, Huazhong University of Science and Technology, 1277 JieFang Avenue, Wuhan 430022, China; ^3^Department of Toxicology, Charles River Laboratories, 6995 Longley Ln, Reno, NV 89511, USA; ^4^Department of Neurology, Tongji Hospital, Huazhong University of Science and Technology, Wuhan 430077, China

## Abstract

**Background:**

The prognosis of patients with hepatocellular carcinoma (HCC) is poor, with 60% to 70% of patients developing recurrence and metastasis within five years of radical resection. Alpha-fetoprotein (AFP) plays a significant role in predicting the recurrence and metastasis of HCC after surgery. However, its role in modulating tumor immunity has not been investigated. Our objective was to examine the effect of AFP on the expression of B7 family and activation of the NF-*κ*B (P65) pathway in HCC.

**Methods:**

We generated human hepatoma SMMC-7721 cell lines with or without recombinant AFP transfection (AFPup and control groups). Colony formation assay, Transwell invasion assay, and wound healing assay were used to detect the function of AFP. Liver cancer xenografts were made in BALB/c nude male mice (*N* = 6 per group). After 28 days of inoculation, the expression of immune genes in the HCC tissues, including PD-L (B7-H1), B7-H3, B7-H4, and P65, was evaluated by quantitative real-time PCR (qPCR) and western blot. In addition, immunofluorescence was used to determine the subcellular localization of the P65 protein, a key factor in the NF-*κ*B pathway. An online HCC patients' dataset was also used to detect the connection between AFP and P65.

**Results:**

Overexpression of AFP could enhance proliferation, invasion, and migration of HCC cells. Both qPCR and western blot results demonstrated that the expressions of PD-L1, B7-H4, and P65 were significantly higher in the AFP group compared to the controls (*P* < 0.05). Immunofluorescence results indicated that the majority of the P65 protein was located in the cytoplasm in the control group but was translocated to the nucleus in the AFPup group. The Spearman correlation coefficient confirms that AFP has a positive correlation with P65 in HCC patients (*R* = 0.33, *P*=0.05).

**Conclusion:**

AFP could enhance proliferation, invasion, and migration in HCC cells. The upregulation of AFP would increase the PD-L1 and B7-H4 mRNA and protein expression in HCC tissues through the upregulation and activation of the P65 protein.

## 1. Introduction

Hepatocellular carcinoma (HCC) is the sixth most common cancer and the third most frequent cause of cancer-related deaths in the world. Its etiology is largely unknown, and it is prone to early metastasis and infiltration to adjacent and distant tissues [1, 2]. Alpha-fetoprotein (AFP) is one of the major proteins in the serum of vertebrate embryos and is highly expressed in HCC. According to the National Comprehensive Cancer Network (NCCN) Clinical Practice Guidelines in Oncology 2019, patients are suspected to develop HCC when they have a serum AFP level > 100 ng/mL or AFP level increases by ≥ 7 ng/mL/month in at least 3 tests. A positive AFP level should prompt computed tomography (CT) or magnetic resonance imaging (MRI) exams regardless of the ultrasonography (US) results [3, 4]. Using the AFP level detection, HCC is the only solid tumor that can be diagnosed by a blood test. AFP has been used as a tumor marker of HCC and plays an essential role in liver cancer screening, diagnosis, monitoring, and prognosis prediction, but its biological functions are still poorly understood [5, 6].

Conventional treatments for cancers include surgery, chemotherapy, and radiotherapy. Immunotherapy and targeted therapy have been gradually introduced to the clinic and recently recognized as the most promising treatment to cure malignant tumors. The healthy immune system detects and clears tumor cells, which is regulated by a large number of immune activators and inhibitors [7]. Tumor cells can express critical regulatory markers of immune checkpoints, such as PD-L1, B7-H3, and B7-H4, to inhibit the body's antitumor immune response and promote tumor immune escape [8].

As AFP is highly expressed in HCC tissues, its involvement in the tumor immune escape mechanisms remains unexplored. In this study, we investigated the effects of AFP on immune-related protein expression and NF-*κ*B pathway (P65) activation, which elucidated the molecular association between AFP expression and tumor immunity.

## 2. Methods

All experimental procedures and protocols were approved by the Institutional Animal Care and Use Committee of Tongji Medical College (Wuhan, China).

### 2.1. Cell Line and Cell Culture

The human HCC cell line SMMC-7721 was obtained from the Chinese Center for Type Culture Collection (CCTCC, Wuhan, China). The cell line was cultured in DMEM (Life Technologies, Beijing, China) supplemented with 10% fetal bovine serum (FBS) (Life Technologies) and maintained at 37°C in a humidified atmosphere with 5% CO_2_.

### 2.2. Genetic Overexpression

The recombinant AFP and negative control lentiviruses were purchased from Shanghai Jikai Genetic Company (Shanghai, China). A single-cell suspension of SMMC-7721 cells was prepared and adjusted to a cell density of 1^*∗*^10^5^/ml. The suspension (1 ml/well) was inoculated into a 6-well plate, with *n* = 3 wells per treatment. After reaching approximately 40% cell confluency, the recombinant AFP or negative control lentiviruses were transfected into the SMMC-7721 cells.

### 2.3. Colony Formation Assay

500 per well of HCC cells from the AFP and control groups were seeded in 6-well plates, respectively. The medium was changed every 3 days. After 10 days, 4% paraformaldehyde (Servicebio, Wuhan, China) was used to fix colonies and 1% crystal violet (Bikeman, Changde, China) was used to stain colonies.

### 2.4. Wound Healing Assay

AFP and control group cells were seeded in 6-well plates with the number of 1 × 10^6^ cells per well. 200 *μ*l plastic pipette tip was used to create wounds for each group and the medium was changed into serum-free medium. Pictures were taken at 0 h, 24 h, and 48 h to measure the migration rate.

### 2.5. Transwell Invasion Assay

Transwell cell culture inserts and BD Matrigel were bought from Corning Costar Corporation (Corning, USA). Both AFP and control groups were seeded in Transwell cell culture inserts 10^^^4 per well with serum-free medium, respectively. The lower chambers were filled with the medium mixed with 20% FBS. 48 h later, cotton swabs were used to clean the cells in the upper surface of the upper chambers. Then, chambers were fixed by 4% paraformaldehyde. 1% crystal violet was used to stain after cells in inserts were fixed. Microscopy was used to count the cells and invasive cells were photographed.

### 2.6. Animal Experiments

SMMC-7721 cells transfected with recombinant AFP or negative control lentiviruses were collected at the exponential phase, processed to cell suspensions, and subcutaneously injected (0.2 ml; 5 × 10^6^ cells) into the right axilla of BALB/c nude male mice (*N* = 6 per group). The longest (*A*) and shortest (*B*) diameters of the tumor were measured every 4 days with a Vernier caliper. The tumor volume was calculated according to the following formula: V (mm^3^) = AB^2^/2. The tumor growth curve was plotted to compare the growth rate of the subcutaneous liver cancer xenografts with or without AFP overexpression. After 28 days of inoculation, the mice were euthanized to isolate the tumors.

### 2.7. qPCR Assay

Total RNA was extracted from the HCC tumor tissues transfected with recombinant AFP or negative control lentiviruses. cDNA was prepared with a PrimeScript® RT Reagent Kit (Takara, Japan). qPCR was performed with a SYBR ®Premix Ex Taq kit (Takara, Japan) on a LightCycler ® 480 system (Roche Diagnostics), according to the manufacturer's protocol. The expression of the target genes was determined by the relative quantitation value against GAPDH expression. The primer sequences are shown in Table 1.

### 2.8. Western Blot Analysis

Western blot was done as previously described [9]. Primary antibodies against AFP, B7-H3, B7-H4, PD-L1 (B7-H1), and P65 were bought from Proteintech (Wuhan, China), and GAPDH was made by Abcam (Cambridge, MA, USA).

### 2.9. Immunofluorescence

Cells isolated from the HCC xenografts transfected with or without AFP were seeded onto 24-well plates and allowed to attach. Next, 4% paraformaldehyde was added for 15 minutes to immobilize cells. After washing 3 times with PBS, 0.25% Triton X was added for 15 minutes on ice to improve the permeability of the cytomembrane. The plate was washed 3 times more with PBS before the addition of goat serum for 30 minutes at room temperature for blocking. After rinsing with PBS, the cells were incubated with the anti-P65 (1 : 50) antibody at 4°C overnight. The primary antibody was removed, and the plate was washed 3 times with PBS. Samples were then incubated with a Cy3-conjugated secondary antibody for 1 hour at room temperature. After washing with PBS, the cellular nuclei were stained with DAPI for 15 minutes at room temperature. High-magnification fluorescent images were obtained using an inverted epifluorescence microscope (Nikon, Japan). The localization of P65 expression was analyzed using ImageJ software (National Institutes of Health, Bethesda, MD, USA).

### 2.10. Clinical Data

Clinical samples were obtained from a HCC proteomic research [10]. The research collected 35 patients' HCC tissues and used proteomic and phosphoproteomic profiling to characterize their gene expressions. This article was published in *Nature* in 2019, and its data had been uploaded to a data portal at http://liver.cnhpp.ncpsb.org/. Researchers could access these data from this open-source online dataset. We used these data to explore the connection between AFP and P65.

### 2.11. Statistical Analysis

Statistical analysis was performed with SPSS® version 23.0. *T* test was used to compare differences between groups. The correlation between AFP and P65 was analyzed using the Spearman test. Differences with *P* ≤ 0.05 was considered as statistically significant.

## 3. Results

### 3.1. AFP Accelerates HCC Cell Proliferation In Vitro

**Figure 1:**
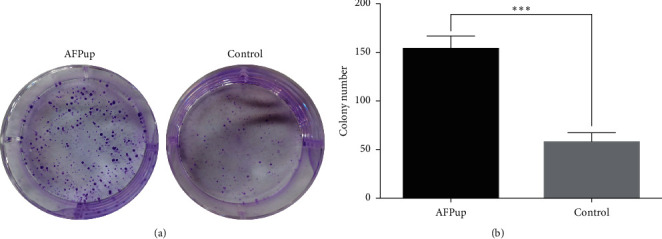
(a) SMMC-7721 cells transfected with or without AFP were seeded in 6-well plates. The number of colonies stained was counted after 10 days. (b) The number of colonies in the AFPup group was significantly more than that in the control group (^*∗∗∗*^ indicates *P* < 0.001).

The colony formation assay results showed that the upregulation of AFP in HCC cells could enhance its ability of proliferation (Figure 1). Colonies in the AFPup group were significantly larger and more than the control group (*P* < 0.001).

### 3.2. AFP Could Enhance Invasion and Migration of HCC Cell Line

**Figure 2:**
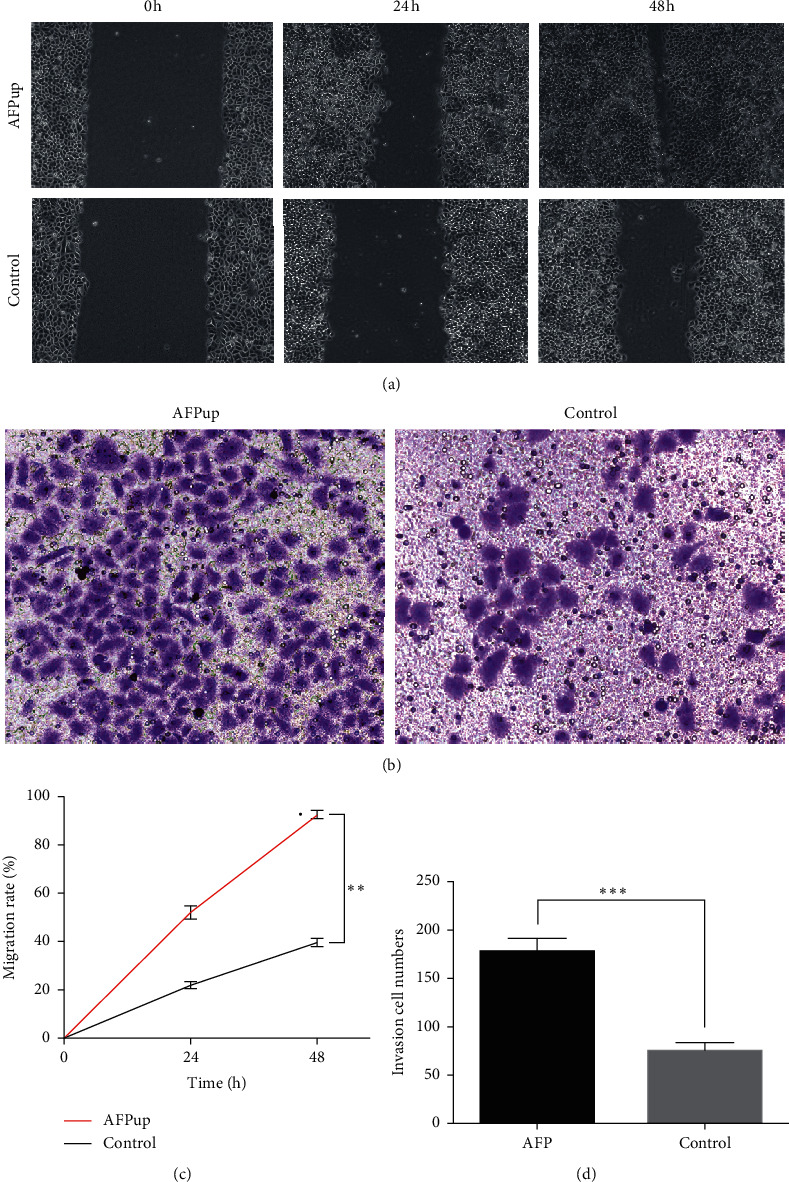
(a, c) In wound healing assay, migration rate in the AFPup group is higher than that in the control group at 24 h and 48h. (b, d) Transwell results showed that more invasion cells could be observed in the AFPup group (^*∗∗*^ indicates *P* < 0.01 and ^*∗∗∗*^ indicates *P* < 0.001).

In wound healing assay, the migration rate in the AFPup group is higher than that in the control group at 24 h and 48 h (*P* < 0.01) (Figures 2(a) and 2(c)). Transwell results showed that more invasion cells could be observed in the AFPup group (*P* < 0.001) (Figures 2(b) and 2(d)). From these results, we have found that upregulation of AFP could enhance the invasion and migration of HCC cells.

### 3.3. AFP Promotes HCC Xenograft Growth in the Nude Mouse Model

**Figure 3:**
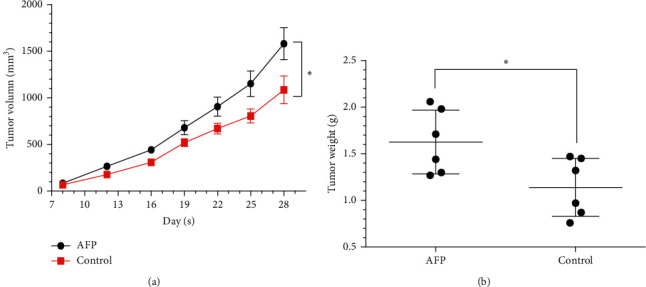
The growth curve of subcutaneous hepatocellular carcinoma xenografts transfected with AFP or negative control lentiviruses in BALB/c nude male mice (*N* = 6 per group). The xenografts in the AFP group were significantly larger and heavier compared to controls (^*∗*^ indicates *P* ≤ 0.05).

The rate of tumor-bearing was 100% in the mice inoculated with the SMMC-7721 cells, and none of the tumor-bearing mice died during the experiment. The growth of the tumor volume was significantly faster in the AFP group compared to the controls. After 28 days of inoculation, the volumes of dissociated xenografts were 1580.50 ± 420.99 mm^3^ and 1085.77 ± 365.35 mm^3^ for the AFP and control groups (*P*=0.02) (Figure 3(a). The weight of the xenografts in the AFP group is 1.63 ± 0.34 g, while that in the control group is 1.14 ± 0.41 g (*P*=0.03) (Figure 3(b)). The xenografts in the AFP group were significantly larger and heavier compared to controls (*P* ≤ 0.05).

### 3.4. Overexpression of AFP Increases the mRNA and Protein Expression of B7-H4 and PD-L1

**Figure 4:**
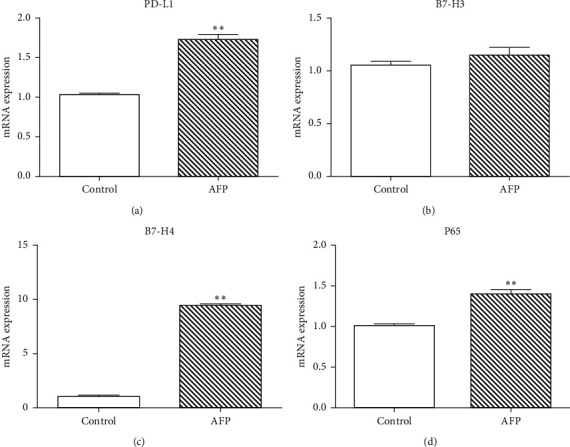
Compared to the control group (xenografts transfected with negative control lentivirus), overexpression of AFP increased the mRNA expression of PD-L1, B7-H4, and P65 in the subcutaneous hepatocellular carcinoma xenografts inoculated in BALB/c nude male mice. There were no differences in B7-H3 mRNA expression between the AFP and control groups (^*∗∗*^ indicates *P* < 0.01).

**Figure 5:**
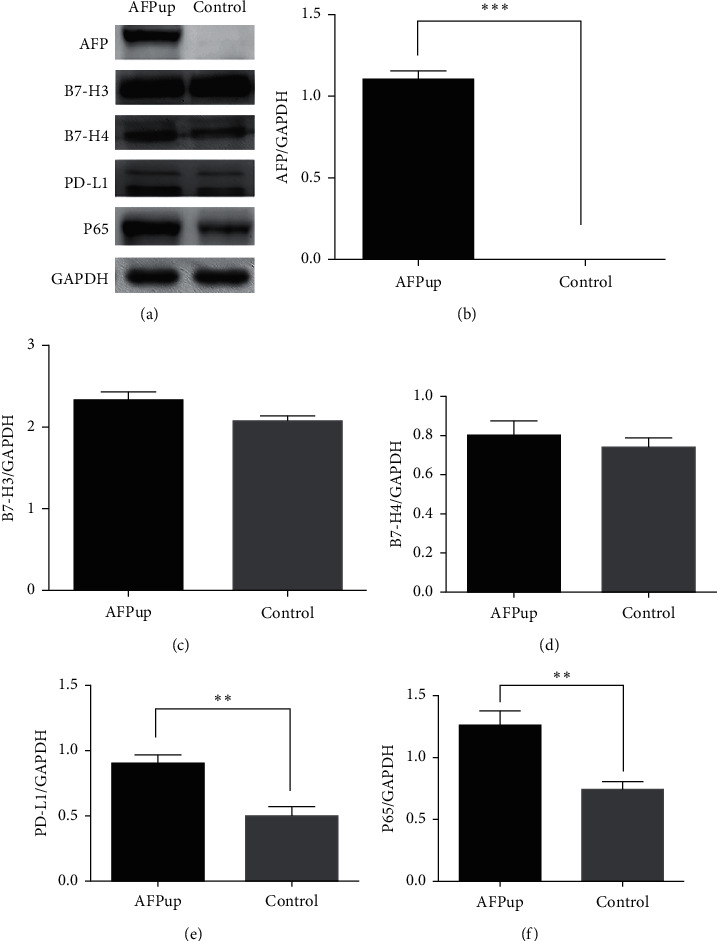
Western blot analysis confirmed that transfection with the recombinant AFP lentivirus increased the AFP expression in the subcutaneous hepatocellular carcinoma xenografts inoculated in BALB/c nude male mice. Compared to the control group (xenografts transfected with negative control lentivirus), overexpression of AFP increased the protein expression of PD-L1, B7-H4, and P65. There were no differences in B7-H3 protein expression between the AFP and control groups (^*∗∗*^ indicates *P* < 0.01 and ^*∗∗∗*^ indicates *P* < 0.001).

qPCR results showed that the mRNA expression of PD-L1 and B7-H4 was significantly higher in the AFP group compared to controls (*P* ≤ 0.05; Figure 4). Consistently, western blot analysis also indicated higher PD-L1 and B7-H4 protein expression in the AFP group than controls (Figure 5). There were no differences in B7-H3 mRNA or protein expression between the AFP and control groups.

### 3.5. AFP Enhances the Expression and Nucleus Translocation of P65

**Figure 6:**
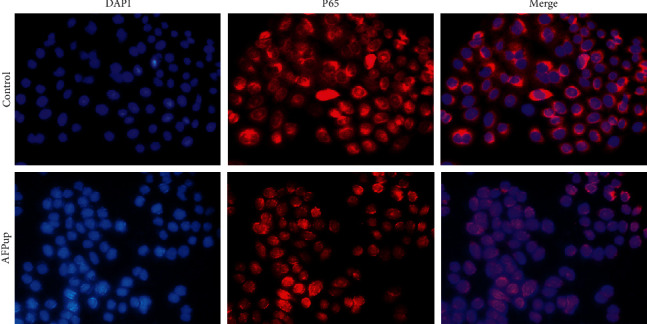
Immunofluorescence analysis revealed that the majority of the P65 protein (red) was in the cytoplasm of cells from the subcutaneous hepatocellular carcinoma (HCC) xenografts transfected with negative control lentivirus and inoculated in BALB/c nude male mice. The P65 protein was translocated in the nucleus (blue) in the cells of the HCC xenografts transfected with recombinant AFP lentivirus.

Both the qPCR and western blot results showed that the expression of P65, a key protein in the NF-*κ*B pathway, was increased in the AFP group compared to controls (Figures 4 and 5). Immunofluorescence analysis revealed that the majority of the P65 protein was located in the cytoplasm in the control group but was translocated to the nucleus in the AFP group (Figure 6).

### 3.6. AFP Has a Positive Correlation with P65 in HCC Patients

**Figure 7:**
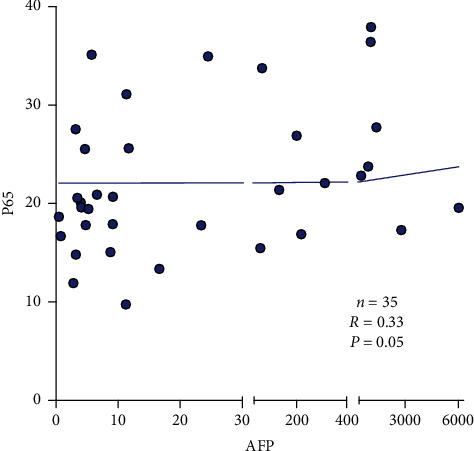
We used open-source online data to test the correlation between AFP and P65. 35 HCC patients were included in the dataset. P65 showed a positive correlation with AFP (*R* = 0.33; *P*=0.05).

We used open-source online data to test the correlation between AFP and P65. 35 HCC patients were included in the dataset [10]. P65 showed a positive correlation with AFP (*R* = 0.33, *P*=0.05) in HCC patients (Figure 7).

## 4. Discussion

At present, immunotherapies targeting immune checkpoints offer promising treatment options for many cancers. T cells are the major immune cell population to fight tumor cells, and their activation is regulated by the innate immune system through positive and negative costimulatory molecules [11]. The B7/CD28 family plays a crucial role in the regulation of T-cell responses by providing costimulatory and coinhibitory signals [12, 13]. Nine members of the B7 family have been documented in the NCBI, and they play important roles in the regulation of the adaptive immune system using Ig/TCR/MHC [14]. According to previous reports, the B7 family is associated with HCC aggressiveness, leading to a poor prognosis [15–19]. Some reports have confirmed that the differential expression of PD-L1 (B7-H1) in HCC patients may lead to different prognosis outcomes, and inhibition of the PD-L1 immunomodulatory pathways may contribute to antitumor effects in HCC [20–22]. B7-H3 triggers inhibitory signals in effector T cells and promotes the progression of hepatitis B virus (HBV) infection, which may lead to the development of HCC [23]. B7-H4 is believed to be involved in the pathogenesis, recurrence, and antitumor immunity of HCC [24, 25], and overexpression of B7-H4 may cause poor overall survival in HCC patients [26].

AFP is mainly present in embryonic hepatocytes, whereas its expression is almost undetectable after adulthood. However, AFP is elevated in almost all liver cancer patients. A large number of clinical studies have reported that HCC patients with high AFP mRNA and/or protein levels in the blood have higher metastasis and postoperative recurrence rates than patients with low AFP levels [27, 28]. In our study, upregulation of AFP could heighten proliferation, invasion, and migration of HCC cells in vitro. Our current study also revealed that AFP increased the expression of PD-L1 and B7-H4, which may enhance the immune checkpoint function and inhibit the antitumor immune response, leading to immune escape. In order to find out the interaction network between AFP and B7 family, we search the PubMed for more information. During our reading, we found an article, which had introduced transcription factors that bound around PD-L1 [29]. After reading more articles, we suggest that P65 may be a factor to control PD-L1 expression [30–33]. Thus, we choose P65 as an object of our study.

P65, also known as Rel-A, is one of the most important proteins in the NF-*κ*B pathway [34]. P65 regulates transcriptional activities and promotes the binding of P50 and DNA [35, 36]. Under normal conditions, NF-*κ*B-related proteins are sequestered within the cytoplasm through direct interactions with the inhibitor proteins. Upon activation, P65 and P50 form a heterodimer and translocate to the nucleus, recruit transcription coactivators, bind to the target DNA elements, and activate the transcription of downstream genes [37,38]. In our study, immunofluorescence results showed that with high AFP expression, the P65 protein was translocated to the nucleus, indicating that the NF-*κ*B pathway was activated in the AFP group. qPCR and western blot analyses revealed the upregulation of P65, as well as PD-L1 and B7-H3, in the AFP group. It suggested that upregulation of PD-L1 and B7-H3 by AFP was mediated by the activation of the NK-*κ*B (P65) pathway in HCC cells. By using online dataset, we also confirmed that the expression of P65 has a positive correlation with the level of AFP in HCC patients.

In conclusion, our results demonstrated that AFP could enhance proliferation, invasion, and migration of HCC cells. It also has the ability to increase the expression of PD-L1 and B7-H4 in HCC xenografts inoculated in nude mice, possibly via activation of the NF-*κ*B (P65) pathway. Therefore, AFP may promote tumor immune escape, leading to HCC tumor growth, metastasis, and recurrence.

## Figures and Tables

**Table 1:** Primer sequences for qPCR analysis.

Gene	Oligonucleotide sequence
P65	(F) 5′-TGCTGTGCGGCTCTGCTTCC-3′
	(R) 5′-AGGCTGGGGTCTGCGTAGGG-3′
PD-L1	(F) 5′-GGTGCCGACTACAAGCGAAT-3′
	(R) 5′-TAGCCCTCAGCCTGACATGTC-3′
B7-H3	(F) 5′-ACCATCACAGGGCAGCCTAT-3′
	(R) 5′-TCCTCAGCTCCTGCATTCTC-3′
B7-H4	(F) 5′-CCCAATCCGAAGTGTCAACT-3′
	(R) 5′-TATCCTGGTGCCCGATAGAG-3′
GAPDH	(F) 5′-GCACCACCAACTGCTTAGC-3′
	(R) 5′-GGCATGGACTGTGGTCATA-3′

## Data Availability

All data generated or analyzed in this study are included in the article.

## Abbreviations

HCC: Hepatocellular carcinoma
AFP: Alpha-fetoprotein
PCR: Polymerase chain reaction
NCCN: National Comprehensive Cancer Network
FBS: Fetal bovine serum
BSA: Bovine serum albumin.
